# Second Trimester Heterotopic Triplet Pregnancy with Intrauterine Twin Pregnancy and Ruptured Interstitial Pregnancy: A Maternal Near-Miss Case Report

**DOI:** 10.1155/2020/5240848

**Published:** 2020-03-11

**Authors:** Charles Nkurunziza, Theogene Rurangwa, Vincent Ngendahimana, Urania Magriples

**Affiliations:** ^1^University of Rwanda, Department of Obstetrics and Gynecology, Rwanda; ^2^Rwanda Military Hospital, Department of Obstetrics and Gynecology, Rwanda; ^3^Yale University School of Medicine, Division of Maternal Fetal Medicine, USA

## Abstract

Heterotopic pregnancy is defined as the occurrence of simultaneous intrauterine and extrauterine pregnancies. It is a rare, potentially life-threatening condition and infrequent in natural conceptions. Here, we report a case of spontaneous heterotopic triplet pregnancy with ruptured cornual ectopic pregnancy and simultaneous twin intrauterine pregnancies at 18 weeks of gestation. The event led to miscarriage of all fetuses from both the ectopic and the intrauterine twin pregnancies.

## 1. Introduction

Heterotopic pregnancy is defined as the occurrence of simultaneous intrauterine and extrauterine pregnancy [1]. It is a rare and potentially life-threatening condition which is infrequent in natural conception cycles with the incidence of 1 in 30,000 pregnancies [1, 2]. Its occurrence rises to 1 : 100 to 1 : 500 with the use of assisted reproductive technologies [1, 2]. We report a case of spontaneous heterotopic triplet pregnancy with an intrauterine twin pregnancy and ruptured cornual pregnancy diagnosed at 18 weeks of gestation. The rupture of the cornual pregnancy resulted in miscarriage of all fetuses from both the ectopic and the intrauterine twin pregnancies.

## 2. Case Presentation

**Figure 1:**
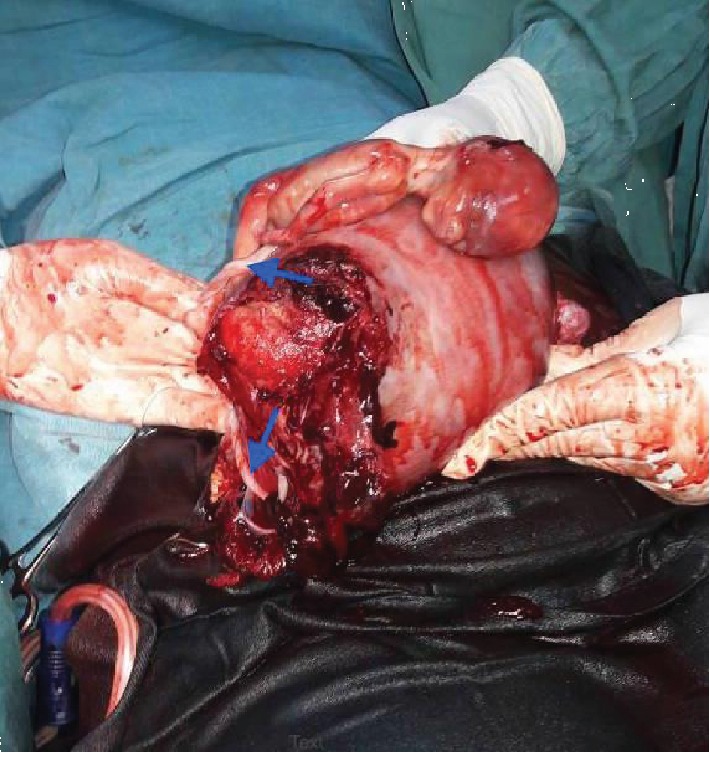
Fetus from the ruptured right interstitial pregnancy alongside the gravid uterus after its exteriorization. The placenta and umbilical cord are still attached to the ruptured site as shown by the blue arrows.

**Figure 2:**
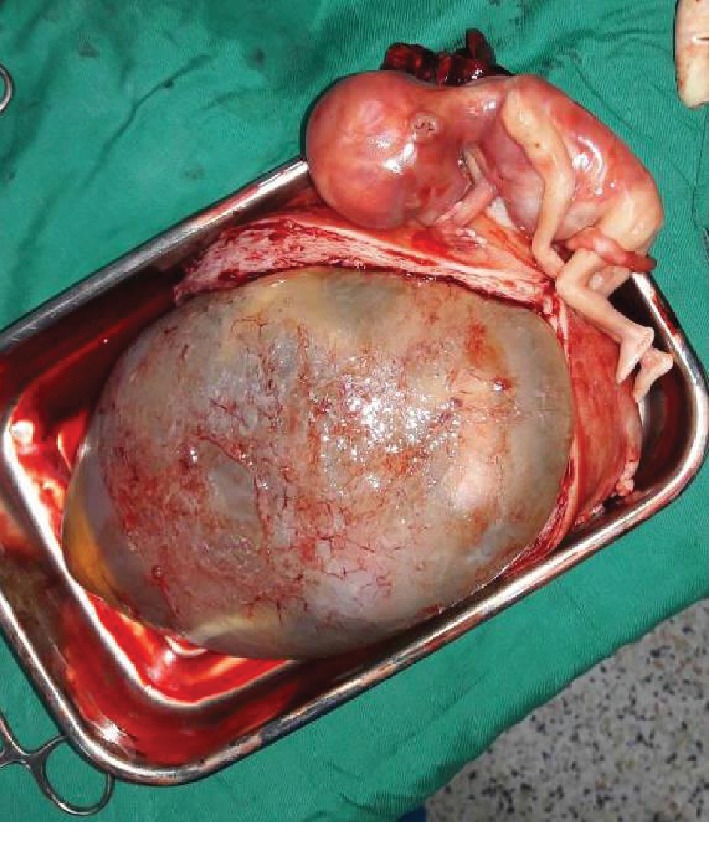
Fetus from ruptured interstitial pregnancy with an open uterus and intrauterine pregnancy membranes still intact.

**Figure 3:**
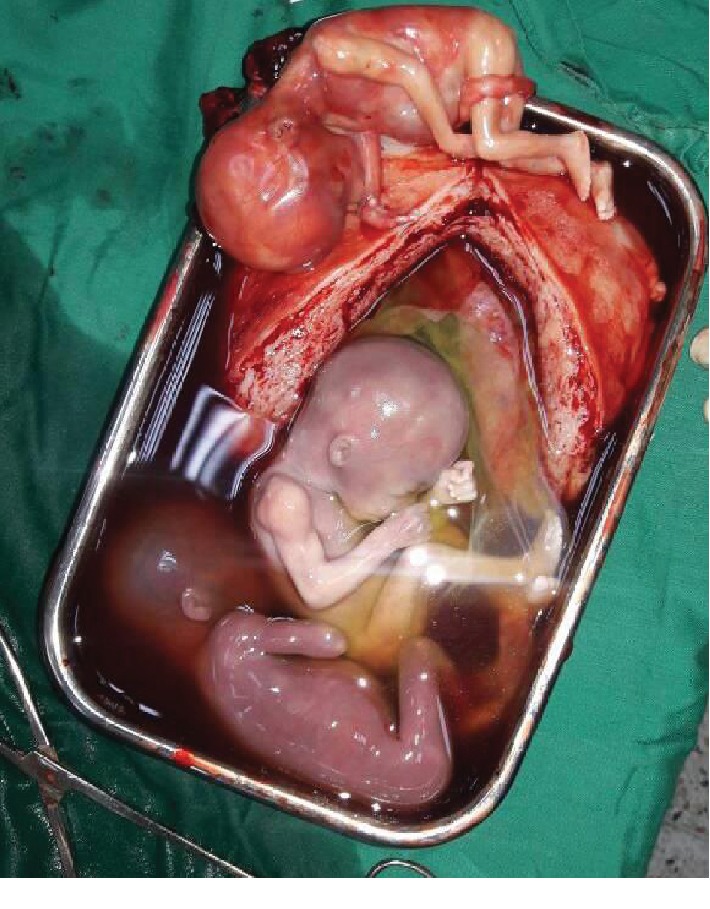
Intrauterine twin pregnancy after rupturing the membranes, with the fetus from the ruptured interstitial pregnancy.

A 34-year-old G6P2122 at 18 weeks of gestation with a history of 2 prior cesarean deliveries was transferred to the Rwanda Military Hospital (RMH) in Kigali, Rwanda, in hypovolemic shock. She had one prior antenatal visit but no ultrasound was done. Her history was also remarkable for pregnancy on IUD contraception. She was seen at a health center with a history of sudden onset severe abdominal pain followed by general body weakness and was referred to a district hospital where ultrasound showed miscarriage of an intrauterine twin pregnancy at 18 weeks of gestation and an additional intra-abdominal fetus dead as well. They suspected uterine rupture and transferred the patient to RMH after initiating IV fluids and transfusion. Travel time was approximately 3 hours, and she reached RMH 12 hours after the onset of symptoms in hypovolemic shock with tachypnea and tachycardia of 144 bpm. She was pale with a distended abdomen and rebound tenderness. Point of care ultrasound showed an intrauterine twin pregnancy with no cardiac activity for both fetuses. A third fetus was seen outside the uterus without cardiac activity and there was free fluid in the abdomen. Results of the full blood count (FBC) at admission came with Hb of 11.6 g/dL, WBC of 28.31 × 10^3^/*μ*L, and Plt of 280 × 10^3^/*μ*L. The patient was immediately taken to the operating room for explorative laparotomy, and consent for possible hysterectomy was signed. Intraoperatively, approximately 4 L of blood and clots was aspirated. A right side interstitial ruptured pregnancy with a dead male fetus in the abdomen with its placenta still attached to the ruptured site was found (Figure 1). The uterus was not reparable because of the extent of the cornual rupture, and a subtotal hysterectomy was performed. Postoperatively, the uterus was opened and a monochorionic-diamniotic twin intrauterine pregnancy was noted (Figures 2 and 3). The patient received blood products intraoperatively and postoperatively. She fully recovered from anesthesia and was discharged from the hospital on her 4^th^ day post operation.

## 3. Discussion

Heterotopic pregnancy is rare in spontaneous natural pregnancy with the incidence of 1 in 30,000 pregnancies, and heterotopic triplet pregnancy with ectopic pregnancy coexisting with twin intrauterine pregnancy is rarer [1, 3]. Risk factors for heterotopic pregnancy are similar to risk factors for ectopic pregnancy and include previous ectopic pregnancy, pelvic inflammatory disease, infertility, in vitro fertilization, previous tubal reconstructive surgery or pelvic surgery, failed contraceptive methods like intrauterine devices and tubal sterilization, and smoking [4–7]. For our case, the identified risk factor was a failed intrauterine device (IUD) as she had conceived on IUD contraception. A high index of suspicion and transvaginal ultrasound are important in timely diagnosis of heterotopic pregnancy especially in spontaneous pregnancies [1]. Early transvaginal ultrasound performed by an experienced sonographer has both high sensitivity and specificity in diagnosing heterotopic pregnancy [8]. The timing of the diagnosis of heterotopic pregnancy is variable from 5 to 34 weeks with the majority being diagnosed between 5 and 8 weeks of gestation [2]. When the diagnosis is made before rupture of the ectopic pregnancy, the prognosis for the intrauterine pregnancy is good [1]. However, timely diagnosis is only possible where the prenatal ultrasound is available, and women seek for antenatal consultation early in pregnancy. In our case, the diagnosis was made late as she was in hypovolemic shock on presentation. The diagnosis of heterotopic pregnancy is not easy but adequate training of primary and secondary care doctors and/or sonographers in early pregnancy ultrasound can help in achieving timely detection of heterotopic or simply ectopic pregnancies [8].

## 4. Conclusion

A high index of suspicion is essential to diagnosis of heterotopic pregnancy especially in spontaneous pregnancies, and clinicians should be aware that confirming an intrauterine pregnancy does not exclude the risk of ectopic pregnancy. Clinicians performing antenatal ultrasound scans should thoroughly scan the pelvis to rule out an extrauterine pregnancy despite having confirmed the intrauterine pregnancy.

Educating women for early first antenatal consultation to confirm the location of pregnancy and improving access to ultrasound in antenatal clinics is the key to saving lives of women from heterotopic pregnancies or ectopic pregnancies burden.

## Abbreviations

BP: Blood pressure
FBC: Full blood count
EGA: Estimated gestational age
g/dL: Grams per deciliter
GCS: Glasgow coma scale
Hb: Hemoglobin
HR: Heart rate
IUD: Intrauterine device
IV: Intravenous
Plt: Platelets
RMH: Rwanda Military Hospital
RR: Respiratory rate
SPO2: Oxygen saturation
*μ*L: Microliter
US: Ultrasound
WBC: White blood cells.
